# Promoting environmental sustainability through school food procurement in low- and middle-income countries: critical reflections

**DOI:** 10.3389/fnut.2025.1687603

**Published:** 2025-12-17

**Authors:** Luana F. J. Swensson, Florence Tartanac

**Affiliations:** Food and Agriculture Organization of the United Nations (FAO), Rome, Italy

**Keywords:** sustainable public procurement, school feeding, environmental sustainability, food systems, low- and middle-income countries

## 1 Introduction

There is a growing attention from policy makers, development partners and donors on the promotion of environmental sustainability through school feeding programmes in Low- and Middle-Income Countries (LMIC) contexts (1–3). School feeding programmes currently reach approximately 466 million children daily and represent an estimated investment of US$84 billion (42), making them a powerful lever for promoting environmental—alongside social and economic—sustainability outcomes and promoting increased access to healthy diets from sustainable food systems. Illustrative of this growing interest are emerging concepts and initiatives such as “Regenerative” (3) and “Planet-Friendly” (1) school meal programmes.

This increased focus builds on previous work that recognizes that, according to choices related to what food to procure, from who, and from what type of production practices, school food procurement—as a key example of public food procurement—can serve as an strategic tool to trigger food systems transformation and promote multiple social (including nutrition), economic and environmental objectives, according to countries policies and national priorities (4–8).

This approach is further supported by existing evidence showing that the strategic use of public food procurement has the potential to contribute to government efforts on climate change mitigation (9, 10), biodiversity conservation (11–13, 43), deforestation (14) and adoption of production practices with no use of synthetic fertilizers or pesticides, such as organic and agro-ecological ones (13, 15–18).

It is also consistent with the United Nations Sustainable Development Goals which recognizes public procurement as a lever for more sustainable production and consumption patterns (Target 12.7). Moreover, it resonates with the broader global discourse (19, 20) and the academic literature on sustainable and green public procurement (21), which highlights the potential of public procurement across different sectors to advance environmental sustainability outcomes.

Although the link between school feeding, public procurement, and environmental sustainability is not new, this connection has received the most policy and scholarly attention in High-Income Countries (HICs), particularly in Europe, where much of the literature is concentrated (22).

In contrast, school feeding and related food procurement policies in Low- and Middle-Income Countries (LMICs)—and the associated literature - have traditionally prioritized social and economic objectives, such strengthening linkages with local agricultural production and supporting smallholder producers (22–25). Existing research draws attention to a persistent disconnecting between school food procurement initiatives and the broader sustainable public procurement debate in these country contexts, emphasizing the need to bridge this gap (4, 7).

With environmental issues increasingly at the forefront of national agendas in LMIC countries, school feeding and food procurement present a valuable opportunity to support countries in meeting both national and international environmental sustainability commitments. Nevertheless, there is still limited discussion—particularly from a LMIC perspective—on the potential challenges to advancing this agenda, especially those linked to public procurement mechanisms.

These include bottlenecks related to (i) defining environmental sustainability for food (22, 26, 27); (ii) operationalizing environmental sustainability objectives through the procurement processes (28–30); and (iii) and managing trade-offs (23, 31–34).

While these challenges are not exclusive to food procurement and may be more commonly addressed in other sectoral sustainable public procurement initiatives, they are rarely examined in the school feeding literature. Addressing these bottlenecks is crucial for advancing the strategic use of school feeding and school food procurement to promote environmental sustainability, particularly from a policymaking perspective. From an academic standpoint, such analysis is equally important for identifying knowledge gaps and highlighting areas in need of further research. This paper seeks to contribute to this effort by offering the school feeding literature a critical reflection on these procurement-related challenges. While school food serves as the central case examined, the insights generated are applicable to broader public food procurement contexts.

## 2 Bottlenecks for promoting environmental sustainability through school food procurement

### 2.1 Defining environmental sustainability for food

Sustainability is a wide and complex concept. While there is a wide consensus that school food should be sustainable, the lack of consensus on what exactly “environmentally sustainable food” is poses important challenges to the regulation and implementation of this policy objective through school food procurement (22, 26, 27).

One important starting point is that environmental sustainability encompasses various dimensions and interconnected pathways. These pathways include energy use; land use and land-use change; biodiversity loss; production and use of fertilizers and pesticides; water use and water pollution; emissions of pollutants from farming and agricultural activities; and disposal of waste (35).

While ideally all these dimensions should be considered together in defining environmental sustainability for food, this is not often the case (33). As highlighted in a literature review by Molin et al., environmental sustainability of food is proxied in most studies by referring (mainly or only) to GHG emissions or assumed benefits for organic and local products (22). These may have a negative impact on policy making, limiting the outreach of school food procurement policies and, in particular, overlooking potential synergies and trade-offs with other dimensions (31).

A decision to only focus on some dimensions of environmental sustainability (such as climate change) may be linked to policy choices and priorities. However, it may also be linked to gaps and related difficulties in how to define and measure the environmental sustainability of food. Difficulties that are often overlooked by the school feeding literature and global debate.

Assessing environmental impacts in food systems involves a large number of decisions, including which environmental impacts and which stages in the life cycle to consider. While a full account should cover as many environmental impacts as possible (and all the different stages in food value chains) current methods and datasets are not equally well developed to consider all relevant environmental impacts (27, 33, 36). Some dimensions (such as energy use and biodiversity) are under-studied while others (e.g., climate change) are frequently analyzed in the literature (27, 37). In addition, studies concentrate mainly on HICs, while there is much heterogeneity among countries and regions, which creates an additional bottleneck (22, 27, 37).

There are also key issues related to the limitations of existing evidence and estimates, which can sometimes lead to misleading information and affect policy formulation and implementation. One example concerns evidence on the carbon footprint of product categories. It is well known, for instance, that the carbon footprint of beef is on average the highest of all food categories. Nevertheless, it is much less well known that this “average” hides much heterogeneity among producers, which may range from around 20 kg CO_2_ eq to over 100 CO_2_ eq per 100 g protein, depending among other things on where and how the food is produced (26, 37). This means that production locations and practices may have a much more important role than is currently considered when addressing environmental sustainability in food procurement, a role that is currently “hidden” in these averages. Similar concerns have been raised by Li et al. (38) regarding estimates related to the carbon footprint of food miles, highlining the importance of utilizing geographically specific footprint information. According to their new methodology, the carbon footprint of food miles were assessed to be 3.5–7.5 times higher than previously estimated, putting the relevance of the location of production for accounting environmental impacts in a new light (38).

While all the authors cited agree that these limitations are linked to the difficulties—and costs involved—in obtaining granular and geographically specific data for true accounting, they also agree that more attention should be paid to this bottleneck and its impact on policymaking. In the case of school food procurement, it is therefore crucial to recognize both the multidimensional nature of environmental sustainability and the challenges in defining and measuring it.

### 2.2 Operationalising environmental sustainability objectives through the procurement process: defining sustainability criteria and verification instruments

Directly related to the challenges involved in defining and measuring environmental sustainability for food is the issue of operationalising it through the procurement process. As is highlighted in the public procurement literature, while vague concepts can facilitate political consensus they are of little use in public procurement, which requires sharply defined criteria for its operationalisation (30). In the case of environmental sustainability and school food procurement, this is an important challenge that is rarely addressed in the school feeding literature.

Indeed, for the pursuit of sustainability objectives in public procurement, these objectives need to be translated into specific criteria that must be accompanied by means of verification. These criteria must be, among others, (i) objective, non-discriminatory and suitable for the aim to achieve; (ii) measurable and quantifiable; and (iii) include a clear evaluation methodology and means of verification.

Defining this type of criteria and related verification instruments is not an easy task, neither in other sectors (39), nor in the specific case of food and environmental sustainability (28–30). One interesting example of this difficulty is the European Union effort to establish voluntary green procurement criteria for the procurement of food and catering services (35). As Shebesta and Casado highlight, it was possible to witness in this process a “harsh struggle to identify certain foods or practices as sustainable, with arguably limited results along some of the sustainability dimensions.” Shebesta and Casado point out that in the revision of the document core criteria were removed from the list of recommendations due to, among others, difficulties and the complexity in identifying suitable efficient criteria that “really bring more sustainability to food procurement.” These include criteria related to integrated production and seasonal foods, despite the latter being the second most used criterion in the EU context (30).

The challenges involved include not only identifying effective criteria but also the choice of verification means, i.e., how in the procurement process it can be verified that the potential supplier complies with the established criteria. Instruments commonly used are labeling and certification schemes, such as organic certifications. Although they may constitute useful instruments, their use presents challenges, especially in LMIC contexts. These challenges include limited coverage (not covering all dimensions of sustainability) and in particular cost, which often affects smallholder producers and may act as a factor excluding access to public market opportunities.

Efforts—such as those by WHO Europe (29) and by ICLEI (40)—are valuable in supporting the design of sustainability criteria and verification instruments. Nevertheless, they remain largely focused on HICs. Further attention and support are still needed in the literature and development partners for LMIC contexts.

### 2.3 Managing trade-offs and synergies

With increasing attention to environmental sustainability in LMIC contexts, it is key to have a better understanding of the potential trade-offs and synergies among the various sustainability outcomes that may be pursued in school food procurement. As highlighted by the literature (31) this is an important bottleneck, considering that the “trade-offs” among social, economic and environmental outcomes have serious implications for policymakers and practitioners in interpreting and implementing school food procurement practices.

Indeed, as is known, not all food that is sustainable from an environmental point of view may be also sustainable from a social point of view, and vice versa. Examples in the EU context show how meeting organic targets often means sourcing imported food through central suppliers, which results in trade-offs between environmental costs (their various dimensions, such as increased food miles) and social and economic costs (31). These trade-offs include the exclusion of smallholder farmers due to certification costs and related challenges (41), and the detachment from the local agriculture production, both aspects particularly relevant for LMIC contexts.

In the case of food, the pursuit of sustainability outcomes must also align with nutrition and health outcomes. Indeed, while the choice to pursue one or multiple social, economic and environmental outcomes may be linked to policy priorities, this is not the case of nutrition and health, which must be priority outcomes for any school feeding programme. Here, more than managing the trade-offs the issue is one of understanding and fostering synergies and policy coherence.

While there is some discussion regarding this in HIC contexts (31, 33) and the broader public procurement literature (34) there is still a gap regarding school food procurement in LMICs. This is particularly critical, considering the unique interplay of social, economic, and environmental outcomes in LMICs. As highlighted in the literature, unlike Europe and many HICs, LMICs have a longstanding tradition of incorporating social and economic considerations into public food procurement, reflecting their national policies and priorities (7, 8, 23). Notable examples include the Home-Grown School Feeding model promoted by the Comprehensive Africa Agriculture Development Programme and various Latin American policies that directly connect school meals with local family farming (24, 25).

As a consequence, the pursuit of new environmental objectives in these country contexts will need to be integrated with these existing social and economic objectives. This necessarily requires managing trade-offs and recognizing the need for an integrated approach to school food procurement. A better understanding of how to manage these trade-offs, foster synergies, and promote policy coherence is therefore key to advancing this agenda.

## 3 Conclusion: final thoughts and the way forward

Pursuing environmental sustainability through school feeding programmes—and their associated procurement mechanisms—offers a valuable opportunity to unlock the full potential of these initiatives and support countries in meeting their environmental commitments. However, several critical bottlenecks remain underexplored in global debates and school feeding academic literature. This paper aimed to highlight and reflect on some of these overlooked challenges.

Based on this reflection, we can affirm that greater attention is needed from policymakers, development partners, and researchers working on school feeding on the challenges related to (i) defining and measuring environmental sustainability for food; (ii) operationalizing environmental objectives through the procurement process and (iii) managing trade-offs and ensuring policy coherence with other existing social and economic objectives.

While these issues may be addressed in other sectors or within the broader public procurement literature, they remain insufficiently explored in the context of school food procurement. This reinforces, as identified in previous studies, the need to strengthen the link between these domains.

To conclude, there is an urgent need for research that further explores these bottlenecks from a multi-disciplinary perspective (including public procurement, environment and nutrition lens) and that is tailored to the specific contexts and realities of LMICs.
